# Effect of carbonic anhydrase on silicate weathering and carbonate formation at present day CO_2_ concentrations compared to primordial values

**DOI:** 10.1038/srep07733

**Published:** 2015-01-13

**Authors:** Leilei Xiao, Bin Lian, Jianchao Hao, Congqiang Liu, Shijie Wang

**Affiliations:** 1 Jiangsu Key Laboratory for Microbes and Functional Genomics, Jiangsu Engineering and Technology Research Center for Microbiology, College of Life Sciences, Nanjing Normal University, Nanjing 210023, China; 2 State Key Laboratory of Environmental Geochemistry, Institute of Geochemistry, Chinese Academy of Sciences, Guiyang 550002, China

## Abstract

It is widely recognized that carbonic anhydrase (CA) participates in silicate weathering and carbonate formation. Nevertheless, it is still not known if the magnitude of the effect produced by CA on surface rock evolution changes or not. In this work, CA gene expression from *Bacillus mucilaginosus* and the effects of recombination protein on wollastonite dissolution and carbonate formation under different conditions are explored. Real-time fluorescent quantitative PCR was used to explore the correlation between CA gene expression and sufficiency or deficiency in calcium and CO_2_ concentration. The results show that the expression of CA genes is negatively correlated with both CO_2_ concentration and ease of obtaining soluble calcium. A pure form of the protein of interest (CA) is obtained by cloning, heterologous expression, and purification. The results from tests of the recombination protein on wollastonite dissolution and carbonate formation at different levels of CO_2_ concentration show that the magnitudes of the effects of CA and CO_2_ concentration are negatively correlated. These results suggest that the effects of microbial CA in relation to silicate weathering and carbonate formation may have increased importance at the modern atmospheric CO_2_ concentration compared to 3 billion years ago.

The evolution of the Earth has been a complex, long-term process^1^. The overall trend in the composition of its surface minerals has involved a constant decrease in silicate and an increase in carbonate minerals. Physical^2^,^3^ and chemical^4^ weathering processes are the main forces driving silicate weathering. In recent decades, the fact that living creatures, especially microorganisms, are involved in mineral weathering has been recognized by a growing number of researchers^5^,^6^,^7^. Microbial weathering results from a combination of many factors^8^ including: bio-mechanical action, the secretion of organic acids, chelation effects, redox reactions, and others. Participation of some active substances during biological weathering makes mineral weathering and enzymatic action more closely linked. Thus, it is worth exploring whether organisms can secrete enzymes to accelerate the weathering of silicate minerals or not and, if they do, how big a role the biological enzymes play in different habitats.

The free metal ions that arise from silicate weathering are involved in the precipitation of carbonates, and this process is accompanied by the fixing of atmospheric CO_2_^9^,^10^,^11^. An important constraint on the formation of carbonates is the concentration of carbonate (CO_3_^2−^) in the metallogenic environment^12^. The acceleration of carbonate formation due to the action of biological enzymes is thus attributed to the increased formation of HCO_3_^−^ and CO_3_^2−^ in the carbonate deposition process. Carbonic anhydrase (CA) was first found in human erythrocytes^13^ and is widely present in animals, plants, and microorganisms. CA shows appreciable CO_2_ hydrase activity (catalytic constants *k_cat_* lie in the range 3.9–8.0 × 10^5^ s^−1^ and kinetic efficiencies *k_cat_/K_m_* are in the range 4.3–9.7 × 10^7^ M^−1^s^−1^)^14^. Thus, CA is capable of catalyzing the reversible hydration reaction, 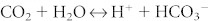, of atmospheric and self-generated CO_2_^15^,^16^.

It has been found that when the environmental CO_2_ concentration changes, organisms may be able to regulate the expression level of the CA gene to adapt to those changes^17^,^18^. For example, the CA gene expression level in mature leaves of young legumes changes following the diversification of CO_2_ concentration: the CA expression level is reduced if the CO_2_ concentration is elevated^19^. *Chlamydomonas reinhardtii* will also increase its CA expression level to take full advantage of CO_2_ when the CO_2_ concentration decreases from 5 to 0.04%^20^. The expression level of their CA genes is increased at lower CO_2_ concentrations. The above research shows that CA may not work at higher CO_2_ concentrations or, perhaps, that it has a more important role in the face of a CO_2_ deficiency. Consequently, it seems more meaningful to express this kind of gene to capture CO_2_ when available levels are low.

When microorganisms grow in environments that have no limits on the availability of elements, many metabolic pathways become very slow (or even stop) to avoid unnecessary material and energy use. A more efficient, economical way is always chosen if they grow in relatively harsh conditions. Expression levels of one, or several, genes will be different according to the difficulty in obtaining nutrition. An anaplerotic role for CA has been proposed which, for example, accounts for the unusual behaviour observed in terrestrial cyanobacteria such as *Nostoc flagelliforme* during hydration–dehydration cycles^21^. The present authors recently showed that the level of CA gene expression in *Aspergillus fumigatus*^22^ and *Aspergillus niger*^23^ is enhanced if the only potassium source available is potassium feldspar (to allow the organisms to obtain potassium more effectively). As it accelerates CO_2_ hydration, CA can promote the generation of H_2_CO_3_, thus promoting weathering of silicate minerals and facilitating the release of K^+^. Moreover, the increased expression of CA by *Bacillus mucilaginosus* favours its survival when the growth environment lacks Ca^2+^ but is rich in calcite^24^. Therefore, an enhanced expression level of the CA gene has a positive impact on microbial growth in environments in which soluble mineral elements are lacking but mineral particles are abundant. The microorganisms not only acquire mineral nutrition but also, at the same time, accelerate the weathering of silicate or calcite. Thus, biological adaptation, with the aid of CA, makes carbon, calcium, and silicon circulation more active.

Carbonate formation is not only an important part of the evolution of surface minerals but also a significant method of fixing atmospheric CO_2_^11^,^25^. Microbial lithification may be the by-product of metabolism^26^,^27^. Some organisms can actively capture CO_2_ and convert it into solid carbonate through CA catalysis^28^. When the CO_2_ concentration is reduced by several orders of magnitude, biomineralization behaviour (in which CA takes part) may affect the growth and even survival of the organism. Previous studies have confirmed that many organisms, such as microbes^29^,^30^, coral^31^,^32^ and animals^33^,^34^, can take advantage of CA's role in CaCO_3_ formation at atmospheric levels of CO_2_. Miyamoto *et al.*, for example, showed that CA from the nacreous layer in oyster pearls is conducive to the formation of CaCO_3_ crystals^35^. Moreover, CA accelerates deposition of minerals and shows greater activity at low CO_2_ concentrations^36^. It has also been reported that CA can contribute to carbonate precipitation at high concentrations^37^. Thus, there is no definitive conclusion as to whether the role of CA is more obvious with a reduction of CO_2_ concentration during CaCO_3_ deposition, or not.

In the work presented here, we use real-time quantitative PCR (RT-qPCR) to study the effect of sufficiency or deficiency in calcium and CO_2_ concentrations on CA gene expression. Inversely, the function of CA in wollastonite dissolution and CaCO_3_ formation, at different CO_2_ concentrations, was investigated using heterologous expression and protein purification. The object of the study is to explore whether the magnitude of the silicate weathering and carbonate formation produced by CA is different at the modern atmospheric CO_2_ concentration compared to that 3 billion years ago.

## Results

### The involvement of CA in wollastonite weathering at the atmospheric CO_2_ level

**Figure 1:**
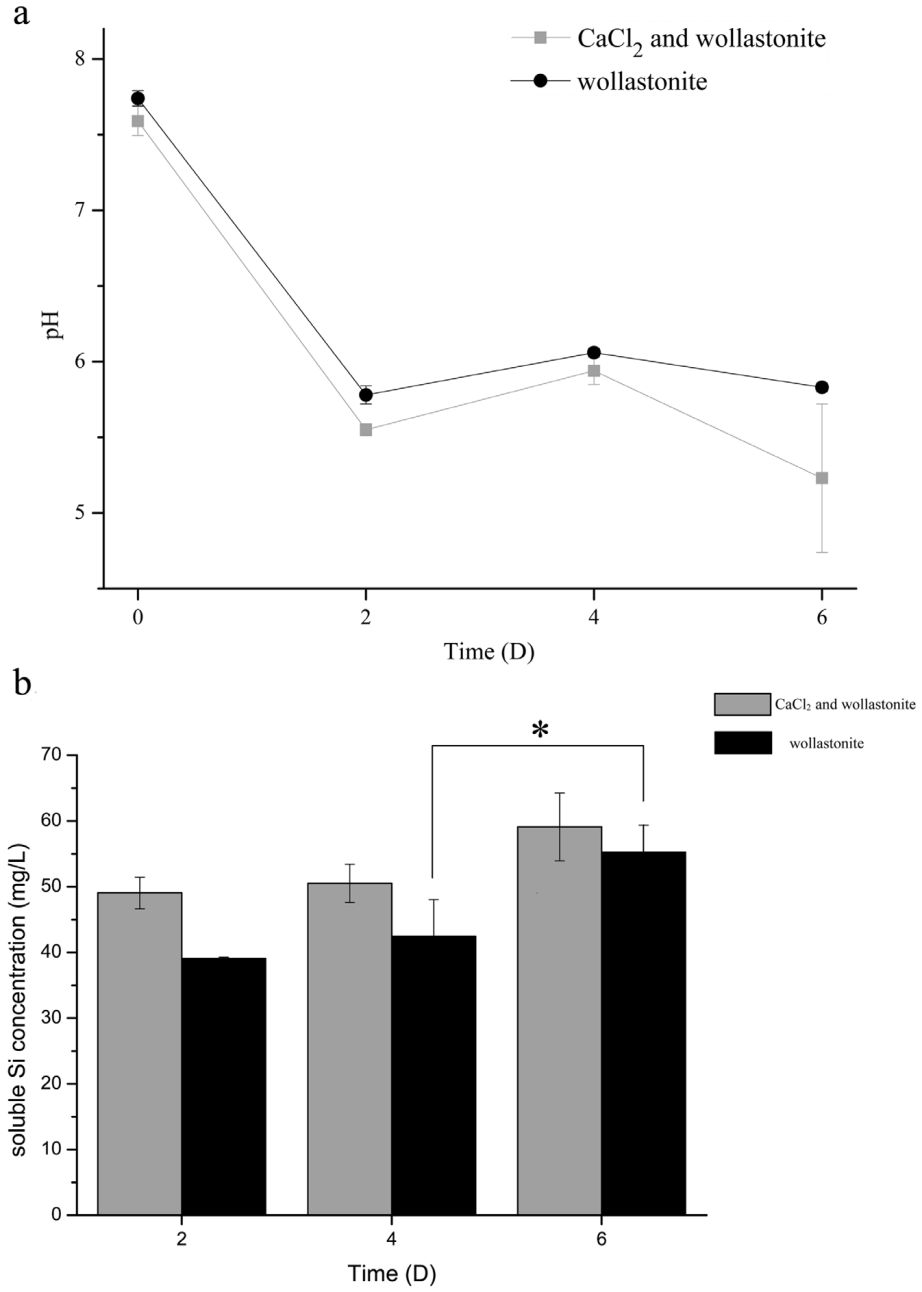
Variation of the pH and soluble silicon concentration of the bacterial cultures at different sampling points (Experiment 1): (a) the pH value at three sampling points; and (b) the concentration of soluble silicon at three sampling points. Bacteria were cultured in a medium with CaCl_2_ and wollastonite as calcium sources (gray bar) or with wollastonite only (black bar). *The results from the two treatments are significantly different (*p* = 0.033).

The results from Experiment 1 are shown in Fig. 1 (see the Methods section for details on the different experiments performed). The trends in the pH variation for the two treatments (i.e. with and without CaCl_2_) are similar (Fig. 1a). There was a sharp initial decrease in pH from day 0 (the primary culture) to day 2. In the days which followed, the pH rose slightly. A significant difference was that a moderate reduction in pH occurred with wollastonite as the only calcium source (compared with that containing CaCl_2_) from day 4 to day 6.

The soluble silicon content (SSiC) of the group with added CaCl_2_ was significantly greater than that in the other group on day 2 (Fig. 1b). However, the differences were not statistically significant (*p* = 0.09 and 0.37, respectively) when the two conditions were compared on days 4 and 6 (Fig. 1b). From day 4 to 6, the SSiC did not increase appreciably (*p* = 0.066) when the medium contained CaCl_2_. However, there was a statistical difference (*p* = 0.033) when the medium only contained wollastonite.

**Figure 2:**
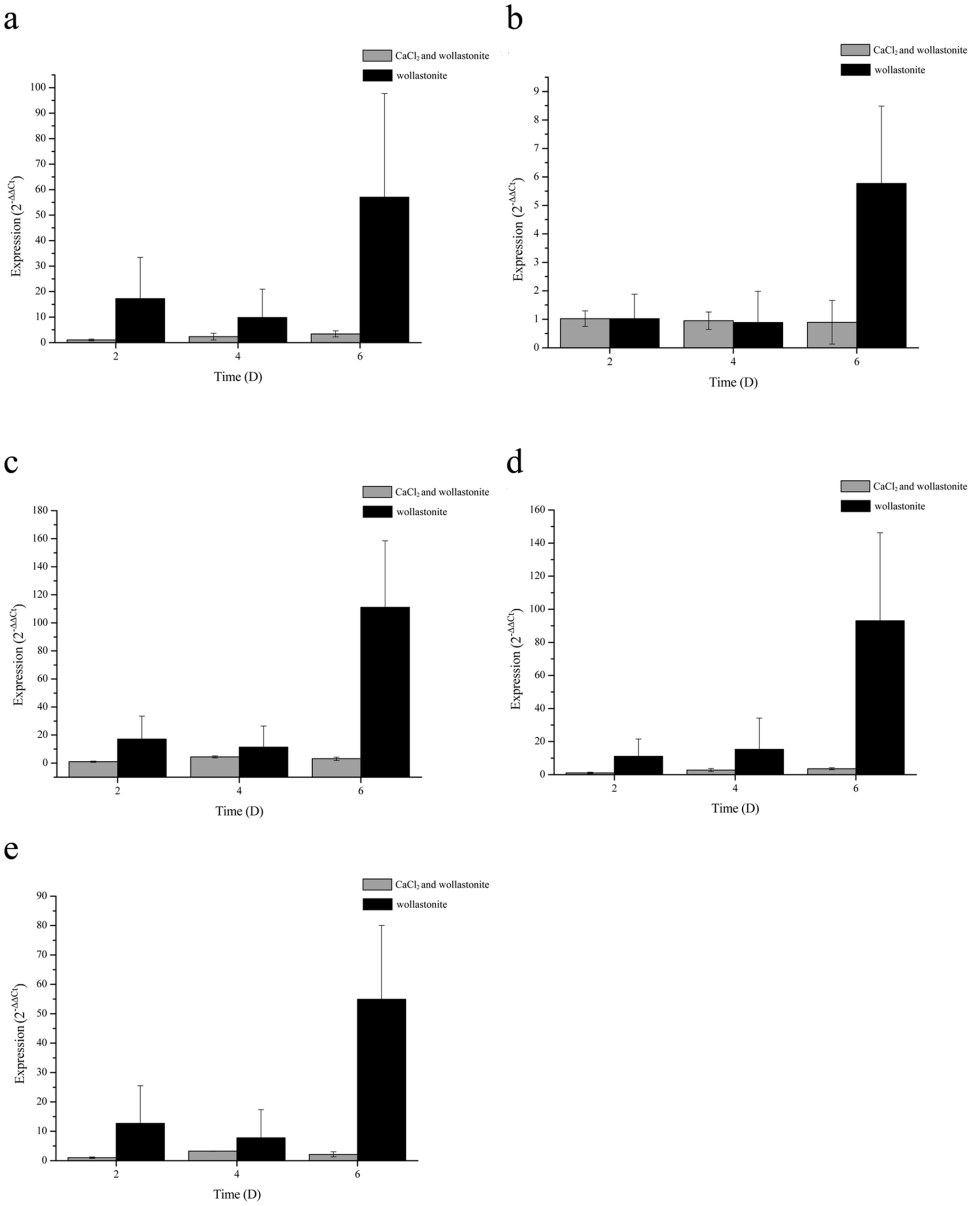
mRNA relative expression levels of five CA genes (Experiment 2). (a), (b), (c), (d), and (e) show the expression of CA1, CA2, CA3, CA4, and CA5, respectively. Gray bars and ‘CaCl_2_ and wollastonite', denote that the calcium sources were CaCl_2_ and wollastonite. Similarly, black bars and ‘wollastonite' denote that the calcium source was wollastonite only. The bacteria were cultured using a concentration of 0.039% CO_2_.

As far as the effect of sufficiency or deficiency in calcium on CA gene expression is concerned (see Experiment 2 in the Methods), none of the CA genes showed markedly different expressions in the two conditions on days 2 and 4 (Fig. 2). The low expression of CA genes and no difference between expressions in the two culture conditions in the early- and mid-growth stages, demonstrates that CA function may not be essential at these points. All five CA genes showed much higher expression levels on day 6 when only wollastonite was present compared to when wollastonite and CaCl_2_ were used (Fig. 2). Furthermore, there was a sharp increase in expression of all genes from day 4 to day 6 when the culture was deficient in calcium. These results indicate that the participation of CA was urgently needed to accelerate wollastonite dissolution in order to provide Ca^2+^ under such conditions.

**Figure 3:**
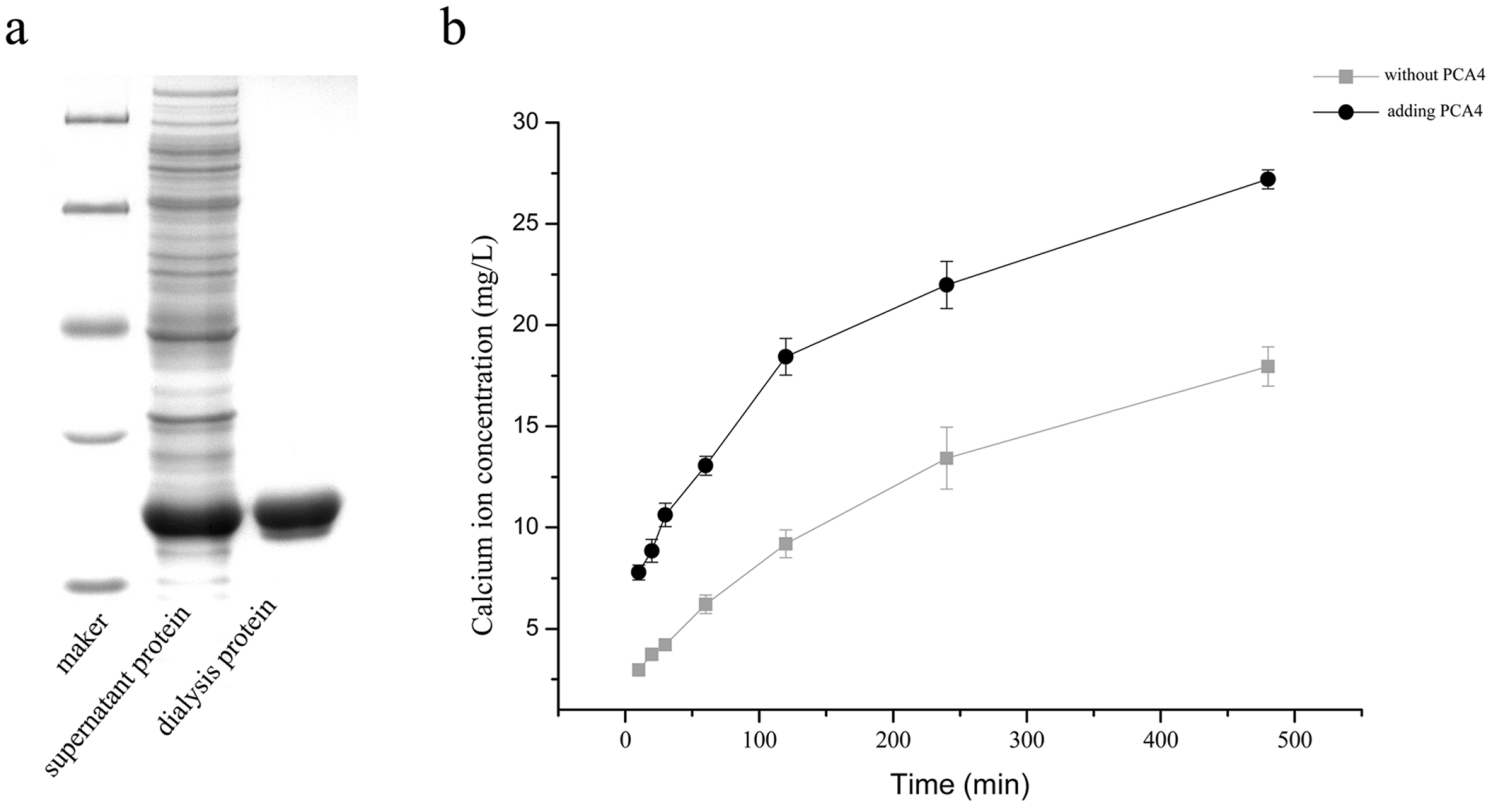
The effect of purified PCA4 on wollastonite dissolution (Experiments 4 and 5). (a) SDS-PAGE analysis of recombination protein (PCA4). The sizes of the protein markers are 116.0, 66.2, 45.0, 35.0, and 25.0 kDa, respectively. (b) Ca^2+^ concentrations at different sampling times, with or without PCA4 in the reaction system, using a concentration of 0.039% CO_2_.

If *B. mucilaginosus* sensed calcium deficiency, significantly increased expression levels of the five CA genes were observed (see Fig. 2). Nevertheless, whether or not a single CA can show any significant effect on the dissolution of wollastonite at the current atmospheric CO_2_ concentration remains unanswered. The current authors tried to find an answer to this by testing the effects of recombinant protein (PCA4) from CA4 gene by heterologous expression on wollastonite dissolution (see Experiments 4 and 5 in the Methods section). The effects of PCA4 on wollastonite dissolution can be seen in Fig. 3. The size of the target protein is consistent with the actual calculated value (28.62 kDa), and no contaminating proteins remained after dialysis (Fig. 3a). The ratio of the dialysis and soluble proteins was about 1:1.225 (gray value), so only a small amount of protein was lost. As can be seen from the dissolution curve (Fig. 3b), the Ca^2+^ concentration, after adding PCA4, was higher everywhere compared to that without it. As the reaction continued, the wollastonite dissolution reached equilibrium. There were only trace amounts of Ca^2+^ released after 8 h. According to the change in the amounts of Ca^2+^ released over time, a pseudo-second-order kinetics model^38^ was constructed to describe the dissolution behaviour of the wollastonite under both conditions (see Table 1). As can be seen from the kinetics equations (Table 1), the value of the dissolution rate *k* after adding PCA4 was 1.402 × 10^−3^ mg g^−1^ min^−1^, and 9.24 × 10^−4^ mg g^−1^ min^−1^ without CA.

### The effect of CO_2_ on CA gene expression and the decreased importance of CA for wollastonite dissolution at high CO_2_ concentration

**Figure 4:**
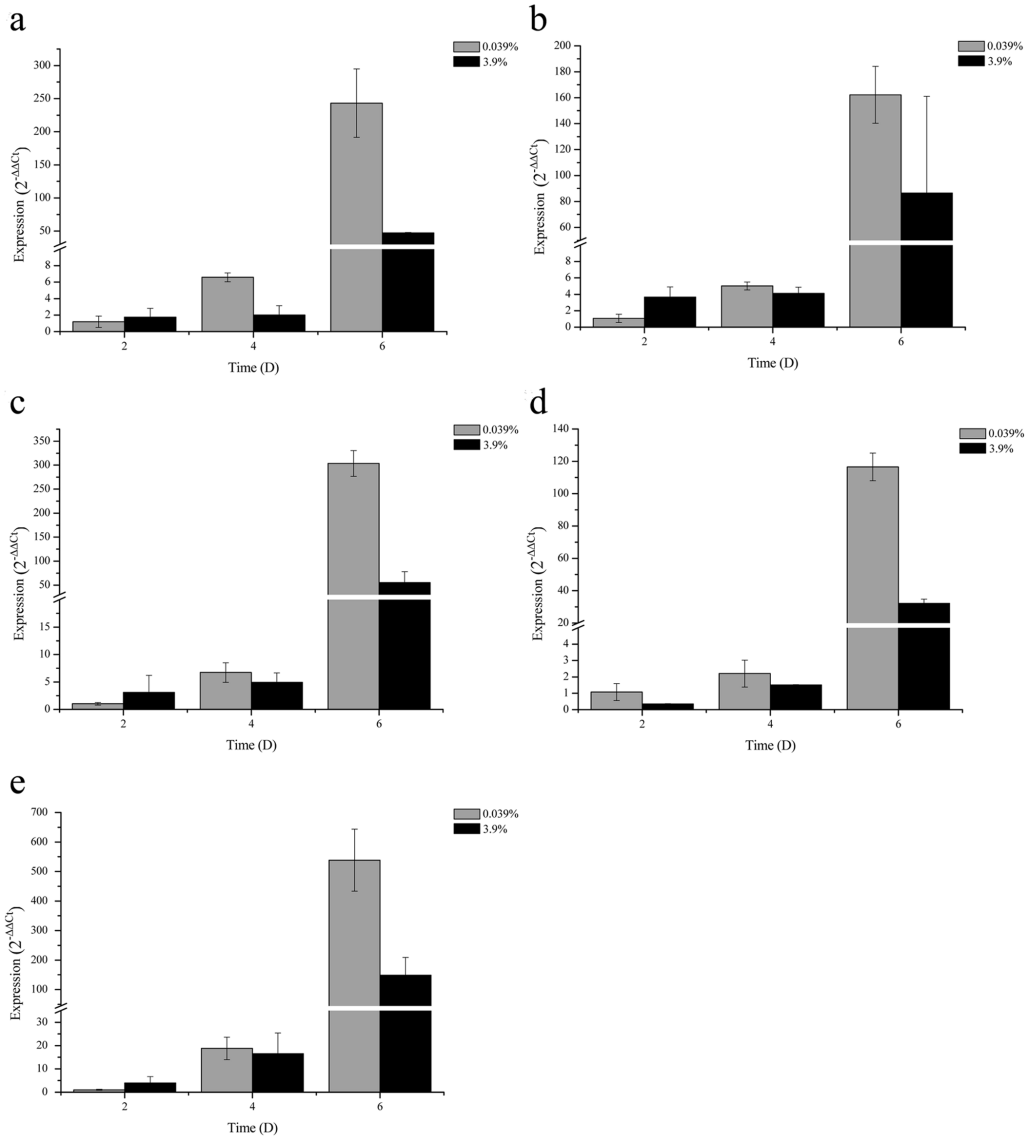
mRNA relative expression levels of five CA genes of *B. mucilaginosus* cultured with wollastonite as the calcium resource using 0.039% and 3.9% CO_2_ concentrations (Experiment 3). (a), (b), (c), (d), and (e) show the expression levels of CA1, CA2, CA3, CA4, and CA5, respectively.

The relative expression levels of the CA genes (displayed in Fig. 4) were significantly different at different sampling times and CO_2_ concentrations (see Experiment 3 in the Methods). The expression of CA3, CA4, and CA5 genes showed no obvious differences on days 2 and 4. This indicates that CO_2_ does not affect the expression of these three genes at this growth stage. However, the expression of CA1, CA3, CA4, and CA5 genes on day 6 were related to CO_2_ concentration. Furthermore, CO_2_ concentration and CA gene expression are negatively correlated. The relative level of expression decreased three- to five-fold when the CO_2_ concentration increased by two orders of magnitude. Additionally, the difference in expression levels obtained by comparing days 6 and 2 reached two orders of magnitude at 0.039% CO_2_. The CA1 gene demonstrated differential expression on day 4. This suggests that the stress of Ca^2+^ deficiency was felt by bacteria at that time. In this case, CA1 was preferred to accelerate the dissolution of wollastonite. This selectivity allows the bacteria to not only adapt to vertiginous environments in a timely manner but also prevents a waste of materials and energy due to superfluous gene expression.

**Figure 5:**
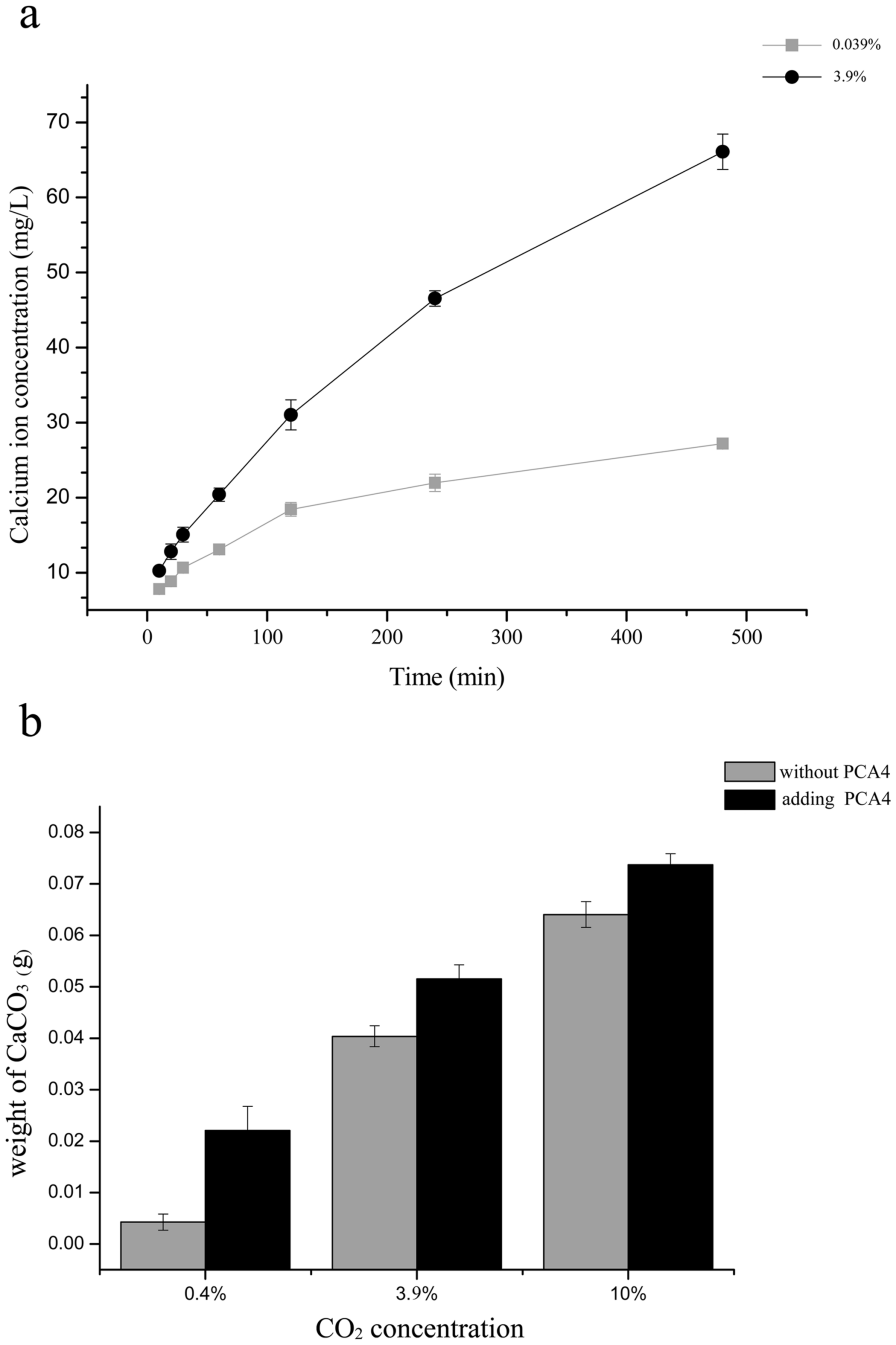
The effect of PCA4 on wollastonite dissolution and CaCO_3_ formation using different CO_2_ concentrations. (a) Ca^2+^ concentration at different sampling times with PCA4 added to the reaction system at atmospheric and 3.9% CO_2_ concentration levels. (b) mineralization in the reaction system without PCA4 (gray bar) or with (black bar) using different CO_2_ concentrations.

To further confirm that the role played by CA in wollastonite dissolution is decreased at higher CO_2_ concentrations, the effect of PCA4 on wollastonite demineralization was determined at both CO_2_ concentrations (see Experiment 5). It can be seen from the observed trends in the amount of dissolved Ca^2+^ (see Fig. 5a) that the dissolution of the wollastonite gradually equilibrated at 0.039% CO_2_ concentration even though PCA4 was added to the reaction system as well. In contrast, Ca^2+^ was released continuously under high CO_2_ conditions. The difference in Ca^2+^ concentration emerged as early as the tenth minute. As the reaction proceeded, the difference increased. Thus, after 8 h, the Ca^2+^ concentration at 3.9% CO_2_ exceeded twice that present at atmospheric CO_2_ levels. These results suggest that CA plays a greater role at lower CO_2_ concentrations than at higher CO_2_ concentrations.

### The impact of CO_2_ on the value of CA in carbonate formation

The results on the impact of CO_2_ on the role of CA in carbonate formation are shown in Fig. 5b. Regardless of whether the reaction system contains PCA4 or not, the CO_2_ concentration is positively correlated with CaCO_3_ production. At any CO_2_ concentration, the CaCO_3_ content (w/w) is significantly different due to the participation of PCA4 (*p* = 5 × 10^−6^, 1.2 × 10^−5^, and 3.3 × 10^−5^ at day 2, 4, and 6, respectively). At 10% CO_2_ concentration, the mass of CaCO_3_ was approximately 0.065 g without PCA4 and more than 0.070 g with PCA4. The proportion of CaCO_3_ that formed due to the participation of PCA4 was about 15%. At low concentrations of CO_2_ (0.4%), the masses were approximately 0.005 g without PCA4 and more than 0.020 g with PCA4. The proportion of the CaCO_3_ formed as a result of the recombinant protein was up by 419%, largely due to the behaviour of the CA. Thus, PCA4 causes a much greater difference in the amount of CaCO_3_ at lower CO_2_ concentrations. The fact that the effect of CA is more remarkable at low CO_2_ concentrations, rather than the opposite, is notable.

## Discussion

Organisms growing on the surfaces of rocks, and thereby causing weathering to occur, are largely there to obtain nutrition^39^,^40^,^41^. Some of the most important inorganic nutrients required for proper cell function are obtained from rocks^42^. In the experiments testing whether wollastonite can induce the expression of CA and whether the weathering behaviour caused by the participation of CA from *B. mucilaginosus* contributes to a certain proportion of the overall mineral weathering effect at atmospheric CO_2_ levels or not, wollastonite was the only available calcium resource when the *B. mucilaginosus* was cultured in media lacking soluble calcium but containing wollastonite. As one group had artificially added CaCl_2_, it is illogical to describe wollastonite dissolution using Ca^2+^ concentration. In view of this, SSiC was used to represent wollastonite dissolution. The number of bacteria on day 2 were about (2.78 ± 0.48) × 10^7^ ml^−1^ and (2.70 ± 0.69) × 10^7^ ml^−1^ with and without CaCl_2_, respectively. We observed a sharp decrease in pH, which may be due to organic acids being secreted by *B. mucilaginosus* in both treatments. Liu *et al.* showed that *B. mucilaginosus* produces organic acids to decompose silicate minerals during its growth, e.g. oxalic acid and citric acid^43^. The overall effect of the bacteria on wollastonite weathering with added CaCl_2_ was stronger, and soon afterwards more soluble silicon was released. Despite the weaker effect without added CaCl_2_, enough Ca^2+^ was released to meet the amount needed for bacterial growth on day 2. Consequently, CA protein may not play an obvious role in wollastonite dissolution and the CA gene expression levels showed no significant differences. As culturing continued, the consumption of organic acids may result in a slight increase in pH. In the culture condition with wollastonite as the only calcium resource, the pressure of calcium deficiency may have been felt on day 6. The wollastonite-only group had a relatively larger bacterial population, (1.21 ± 0.11) × 10^9^ ml^−1^, and less Ca^2+^ than the group containing CaCl_2_ (117.35 ± 10.62 mg/L). A single unit of Ca^2+^, which bacteria were able to gain, was much lower in the group without CaCl_2_. In this case (in the medium without CaCl_2_), the demand of *B. mucilaginosus* for soluble calcium may be stronger. The RT-qPCR results show that the expression of all the CA genes were up-regulated by two orders of magnitude from day 4 to day 6 when wollastonite was the only calcium resource. The consistency between this increase in CA gene expression and wollastonite dissolution (Fig. 1b, from day 4 to 6) suggests that CA plays a role in the dissolution of wollastonite at atmospheric CO_2_ concentrations. The dissolution of wollastonite consumed part of the H^+^ produced by CO_2_ hydration and generated a certain amount of HCO_3_^−^: 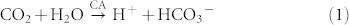

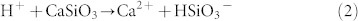


The overall change can be written: 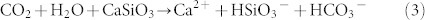
Therefore, the extent of the pH decrease was lower than that with CaCl_2_ and wollastonite as calcium resources (Fig. 1a). This is due to the production of HCO_3_^−^ and consumption of CO_2_ in the medium. There are two aspects to the facilitation of wollastonite dissolution by increasing the expression level of CA: (i) The amount of H^+^ is an important factor if the bacteria is to obtain adequate Ca^2+^ from wollastonite dissolution;(ii) CO_2_ hydration can produce HCO_3_^−^, which is an important substrate for many fundamental biological pathways such as: gluconeogenesis, lipogenesis, ureagenesis, pyrimidine synthesis, and synthesis of several amino acids^44^. CA can participate in the formation of malonyl-CoA, which is catalyzed by acetyl-CoA carboxylase, with bicarbonate and acetyl-CoA as the substrate^45^. Therefore, CA is an important regulator of fatty acid metabolism. Synthesis of fatty acids helps to improve membrane fluidity, which has a certain effect on the efficiency of nutrient acquisition^23^.

Wollastonite dissolution proceeds according to the reaction: 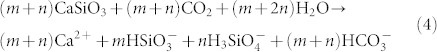
It can be seen from the stoichiometry of this equation that each mole of Ca^2+^ released consumes one mole of CO_2_, which means the relationship that holds between the quantity of Ca^2+^ released and CO_2_ consumed during the process of wollastonite dissolution is: 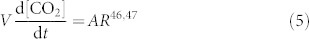
46,47Here *V*, [CO_2_], and *A* represent the solution volume, CO_2_ concentration, and the area of the mineral surface, respectively; *R* is the flux of Ca^2+^ from the wollastonite surface. Thus, for a given volume of solution and mineral surface area, release of Ca^2+^ is proportional to the consumption of CO_2_. From the results of fitting the data to a pseudo-second-order kinetic equation, it is apparent that the *k* values, after adding PCA4, were higher than those without PCA4. This further confirms that CA has a significant role in promoting dissolution of wollastonite at 0.039% CO_2_ concentration. Therefore, enhancement of the CA expression level is an effective way to promote weathering of minerals in order to get the desired inorganic nutrients when the microorganism grows where the CO_2_ concentration is low. This behaviour of the bacteria, to a certain extent, also accelerates the weathering of silicate minerals. The involvement of CA in the demineralization of silicate minerals has also become a recognized part of global biogeochemical cycles.

An increase in CA gene expression level is advantageous to the microbe's survival chances in soluble-calcium deficient environments. However, does CA have a significant accelerating effect on mineral dissolution at high CO_2_ concentrations? The proportion of CO_2_ in the Earth's primordial atmosphere was up to 10%^48^. Previous studies have shown that bacteria can grow in the presence of a CO_2_ concentration of 5%^49^,^50^ and even 10%^51^. In our experiment, the wollastonite underwent dissolution to varying extents at two different levels of CO_2_ concentration (Fig. 5a). The low saturation level resulted in a reduction in dissolution rate and the process gradually reached dissolution equilibrium at 0.039% CO_2_ concentration. In contrast, at 3.9% CO_2_ concentration, the solution had a relatively high saturation level and the dissolution rate remained essentially unchanged as CO_2_ continuously dissolved in the reaction. Therefore, the CO_2_ primarily affected wollastonite dissolution and the function of the CA was not obvious in a sustained high-CO_2_ partial pressure environment. During the early appearance of life (3 billion years ago), silicate weathering mainly occurred due to physical and chemical effects — the contribution of CA to silicate weathering at this time may have been minimal. Atmospheric CO_2_ concentrations gradually decreased (by more than two orders of magnitude) during the process of terrestrial evolution. This implies that the current expression level of biological CA is far higher now than it was in the period during which life emerged. Consequently, the participation of CA in silicate weathering may be much higher now than it was three billion years ago. This means that CA played an increasingly important role in the evolution of the Earth.

Whether it is physical, chemical, or biological weathering that affects the silicate minerals, the process is always accompanied by a release of metal ions. Some can react with HCO_3_^−^ or CO_3_^2−^ in aqueous solution to revert to a solid form^25^. This is also the basic process governing both silicate weathering and carbonate formation. Mineralization experiments have shown that the highest amount of CaCO_3_ occurs under 10% CO_2_ and yet the required HCO_3_^−^ or CO_3_^2−^ during this mineralization mainly arises from spontaneous CO_2_ hydration. The role played by CA in the formation of CaCO_3_ crystals is only responsible for a small proportion of them. Although bacterial CA may have been helpful in promoting the formation of carbonate more than 3 billion years ago^25^, the role might have been negligible because of the small amount of biomass and high CO_2_ concentration. As the atmospheric CO_2_ concentration decreased, non-enzymatic CO_2_ hydration reactions would have become relatively weak. However, the involvement of CA, to some extent, compensated for this reduced rate. When applying CA to capture atmospheric CO_2_, the enzyme efficiency required to accelerate CO_2_ capture increases as the partial pressure of the CO_2_ decreases^52^. The least amount of total mineralization was found at the minimum concentration of CO_2_, but the difference in the amount of CaCO_3_ was maximized at that point. This suggests that CA is more significant at lower CO_2_ concentrations. To some degree, the participation of CA mitigates the reduced rate of carbon fixation and carbonate formation due to the decrease in CO_2_ concentration.

In summary, CA gene expression is negatively correlated with the ease of obtaining soluble calcium and CO_2_ concentration. Moreover, considering the importance of the effect of purified CA on wollastonite dissolution and CaCO_3_ precipitation, the magnitude of the effect of CA is significantly weakened at higher CO_2_ concentrations. In view of the value of the effect of CA at the current atmospheric CO_2 _concentration and that 3 billion years ago, the results suggest that the role of microbial CA may have become increasingly more apparent and important as terrestrial surface rocks have evolved.

## Methods

### Minerals

The wollastonite, Ca_3_(Si_3_O_9_), used in the present study was provided by the State Key Laboratory of Environmental Geochemistry, Institute of Geochemistry, Chinese Academy of Sciences (Guiyang, China). The mineral was crushed and washed according to the method described by Daval *et al*^53^. Briefly, the crushed wollastonite powder was washed using absolute ethanol and sterilized ultrapure water (18.2 MΩ cm^−1^) to eliminate the fine dust resulting from the grinding procedure. Analysis using X-ray diffraction showed that the wollastonite powder contained only trace amounts of calcite and quartz.

### Experiment 1 – Effects of *B. mucilaginosus* on wollastonite dissolution

*B. mucilaginosus* was cultured in a nitrogen-containing medium with different calcium resources to test the effect of *B. mucilaginosus* on wollastonite dissolution (see Table 2). The composition of one kind of medium per litre was as follows: sucrose 10.0 g, (NH_4_)_2_SO_4_ 1.0 g, CaCl_2_ 0.44 g, wollastonite 1.16 g, MgSO_4_ 0.5122 g, KCl 0.1 g, and Na_2_HPO_4_·12H_2_O 2.507 g. The other medium consisted of the same components except for omission of the CaCl_2_. As far as possible, to avoid adsorbed metals and cell exudates being introduced into the medium during inoculation, a seed solution was used according to the description given by Fein *et al.*^54^ (with a little modification). Briefly, the bacteria were cleaned using sterilized ultrapure water (SUW), sterilized HNO_3_ (1 M), SUW, sterilized EDTA (0.001 M), and SUW, respectively. Finally, the precipitate was suspended using 20 ml of SUW and used as seed liquid for inoculation. This operation removes as much inherent calcium as possible. Meanwhile, control media were inoculated with deactivated bacteria to eliminate interference from abiotic factors or mineral dissolution only in the medium. The ratio of seed solution to medium was about 1:10 (v/v). The culture conditions were set at 30°C and 130 rpm at the current atmospheric CO_2_ concentration (0.039%). The pH value of the culture solution was tested at set sampling times (2, 4, and 6 days) using a pH-meter (METTLER-TOLEDO SevenEasy S20). The number of bacteria was counted using a microscope (Zeiss Axio Imager A1, Zeiss, Germany). Moreover, some of the culture solution (15 ml) was then centrifuged (10397 *g*, 4 °C, 30 min) using a centrifuge (Sigma 3 k30) and 5 ml of the supernatant were collected. The remaining liquid was discarded. The precipitate was re-suspended using 10 ml of 1 M ammonium acetate, broken (a minute at a time for a total of three times) using an ultrasonic cell disrupter (Sanyo Soniprep150), and cleaned ultrasonically for 30 min. Ammonium acetate solution was added again to make the total volume up to 15 ml. The solution was mixed and centrifuged (10397 *g*, 4 °C, 30 min) and the supernatant collected. The two kinds of supernatant were mixed in equal volumes and the concentrations of Ca^2+^ and SSiC were detected using ICP–AES (Thermo IRIS Intrepid II XSP). A two-tailed *t*-test was performed using STATISTICA 6.0 software. The data met the assumptions of the test. The mean and its standard deviation were calculated based on three independent experiments.

### Experiment 2 – Effects of calcium resources on CA gene expression

Five CA-related genes [Gene IDs: 12734710 (CA1), 12739330 (CA2), 12735171 (CA3), 12735237 (CA4), and CP003422 region: 5453463–5454707 (CA5)] were annotated in the *B. mucilaginosus* K02 genome. To test the degree of difficulty (or ease) of acquiring Ca^2+^ on CA expression, the same culture conditions were used as in Experiment 1. After 2, 4, or 6 d culturing time, the bacteria were centrifuged (11500 *g*, 4 °C, 1 min). The supernatant was discarded, and the collected cells frozen in liquid nitrogen. Total RNA was then extracted (using an Invitrogen kit in accordance with the manufacturer's instructions) and reverse transcribed into cDNA. The correct RT-qPCR reaction conditions were adopted in accordance with the manufacturer's instructions (SYBR® Premix Ex Taq^TM^ (TliRNaseH Plus), TaKaRa). As an internal reference, 16S rRNA was used (see Table S1). After optimization by testing different primers, a single melting temperature was determined for each of the six pairs of primers, 85.8, 88.2, 87.8, 85.7, 84.8, and 85.5 °C, respectively. The *Ct* value was recorded for subsequent analysis (when the fluorescent signal of each reaction tube reached a set threshold, the number of reaction cycles involved gives the *Ct* value). The mean of *_ΔΔ_Ct* was set to zero on the second day when the bacteria was cultured using wollastonite and CaCl_2_. The relative expression level (REL) was then calculated using the following formula: 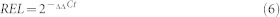.

### Experiment 3 – Effects of CO_2_ concentration on CA gene expression

The CO_2_ concentration was set to either 0.039% or the higher CO_2_ concentration (3.9%) to determine the effect of CO_2_ concentration on CA gene expression (see Table 2). In this experiment, the calcium resource in the medium was only wollastonite. The culture conditions were the same as in Experiment 1. Bacteria collection, RNA extraction, reverse transcription, and the RT-qPCR experiment were carried out as in Experiment 2. The mean of _ΔΔ_*Ct* was set to zero on the second day at 3.9% concentration. REL was then calculated using Eq. (6).

### Experiment 4 – Construction of the heterologous expression vector and induction expression and purification of recombinant protein

The construction of engineered *E. coli* in which five kinds of CA can be expressed was reported previously^24^. The engineered *E. coli*, which over-expresses CA protein from transcription and translation of the CA4 gene referred to as PCA4, was used in the present study. Our recently published research showed that PCA4 had the best solubility and activity compared to four other proteins^24^, and so it was selected for use in this study. Briefly, the CA4 gene was amplified using PCR and then two kinds of restriction endonuclease (Kpn I and Hind III) were introduced using the relevant primers. PCR products and plasmid pET30a were both digested using Kpn I and Hind III and then linked to construct the expression vector. Recombinant plasmids were introduced into *E. coli* BL21 to form recombinant bacteria. Protein was produced by the induction of a final concentration of 1 mM IPTG. After induction, over-expressed PCA4 was obtained using ultrasonication. As there is impure protein mixed with the PCA4, the mixed proteins were purified using Ni-NTA agarose (QIAGEN) in accordance with published research^37^. Shortly after, the mixed proteins were loaded into the Ni-NTA agarose. Then, washing with a buffer (50 mM NaH_2_PO_4_, 300 mM NaCl, 40 mM imidazole, pH 8.0) and elution buffer (50 mM NaH_2_PO_4_, 300 mM NaCl, 250 mM imidazole, pH 8.0) was carried out to remove impure proteins and collect the targeted proteins (PCA4). The eluent containing target proteins was dialyzed twice in dialysate (100 mM tris-sulfate 100, pH 8.0) for 16 h in total. The complete process of protein purification and dialysis was carried out at 4 °C. SDS-PAGE (12.5% polyacrylamide) was used to analyze the target protein as described by Laemmli^55^ with a little modification. Proteins were stained using Coomassie brilliant blue R-250 and decolouration was performed until the band appeared clear. The “gray value” of the proteins were calculated using Photoshop software and used to represent its content.

### Experiment 5 – The effect of CA on wollastonite dissolution

Ultrapure water (49 ml) was added to an Erlenmeyer flask containing 0.116 g of wollastonite; three replicates were tested. Then, 1 ml of ultrapure water and the same amount of PCA4 were rapidly added to the flasks at 35 °C and 130 rpm. Samples were collected at 0, 10, 20, 30, 60, 120, 240, and 480 min (the remaining samples were discarded after each sampling time). The liquid was filtered using a 0.45 μm filter membrane. The concentration of Ca^2+^ was determined by titration using ethylenediaminetetraacetic acid disodium salt (EDTA–Na_2_). To explore whether the importance of the role of CA in wollastonite dissolution at high CO_2_ concentration was similar, the same operation was carried out at 3.9% CO_2_ concentration. To analyze the data, a two-tailed *t*-test was used. The data presented is the mean (along with the standard deviation) of three independent experiments.

### Experiment 6 – Mineralization reaction under different CO_2_ concentrations

A 10 ml (0.2 M) portion of Tirs–HCl (pH 9.0) was mixed with an equal volume of CaCl_2_ (0.2 M) in a clean, sterile Petri dish. Then, 1 ml of ultrapure water and an equal volume of PCA4 were added to the reaction system at 35 °C and rotated at 80 rpm under three different CO_2_ concentrations (0.4%, 3.9%, and 10%). There were three independent replications of each treatment. After 20 min, the supernatant was discarded and the sample dried overnight at 65 °C. The residual crystalline CaCO_3_ was weighed. The dry weight of 1 ml of PCA4 solution is only a few micrograms, so it is negligible in relation to the weight of the CaCO_3 _formed. The statistical approach used to analyze the data was the same as that described above.

## Author Contributions

B.L. and L.X. wrote the main manuscript text; B.L., C.L., L.X. and S.W. designed the experiments; J.H. and L.X. carried out the experiments, and L.X. prepared figures 1-5. All authors reviewed the manuscript.

## Supplementary Material

Supplementary Information Table S1

## Figures and Tables

**Table 1:** The fits of the wollastonite dissolution data to a pseudo-second-order kinetics model

Group	Model	Equation	*q_e_* (mg g^−1^)	*k* (mg g^−1^ min^−1^)	*R*^2^
Without PCA4	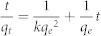	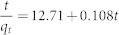	9.225	9.24 × 10^–4^	0.959
With PCA4		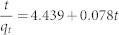	12.67	1.402 × 10^–3^	0.986

**Table 2:** The experimental design used in this study

Experiment	purpose	experimental strain and enzyme	calcium resources	CO_2_ concentration (%)
1	effects of *B. mucilaginosus* on wollastonite dissolution	*B. mucilaginosus*	wollastonite/wollastonite and CaCl_2_	0.039
2	effects of sufficiency or deficiency in calcium on CA gene expression	*B. mucilaginosus*	wollastonite/wollastonite and CaCl_2_	0.039
3	effects of CO_2_ concentration on CA gene expression	*B. mucilaginosus*	wollastonite	0.039/3.9
4	construction of the heterologous expression vector, induction expression and purification of recombinant protein	*E. coli* and enzyme	_____	0.039
5	effects of PCA4 on wollastonite dissolution	enzyme	wollastonite	0.039/3.9
6	effects of PCA4 and CO_2_ concentrations on CaCO_3_ precipitation	enzyme	CaCl_2_	0.4/3.9/10
