# Supplementary material for: Effect of carbonic anhydrase on silicate weathering and carbonate formation at present day CO2 concentrations compared to primordial values
Source: Sci Rep. 2015 Jan 13;5:7733. doi: 10.1038/srep07733 (PMC4291579; doi:10.1038/srep07733)
Supplement: Supplementary Information — Table S1 [file srep07733-s1.pdf]

Effect of carbonic anhydrase on silicate weathering and carbonate formation at present day CO_2 concentrations compared to primordial values

Leilei Xiao[1], Bin Lian[1※], Jianchao Hao[1], Congqiang Liu[2] and Shijie Wang[2]

1. Jiangsu Key Laboratory for Microbes and Functional Genomics, Jiangsu Engineering and Technology Research Center for Microbiology, College of Life Sciences, Nanjing Normal University, Nanjing 210023, China

2. State Key Laboratory of Environmental Geochemistry, Institute of Geochemistry, Chinese Academy of Sciences, Guiyang 550002, China

※Corresponding author Bin Lian, College of Life Sciences, Nanjing Normal University, Nanjing 210023, People's Republic of China

Phone: +86 025 85891050 fax: +86 025 85891050 e-mail: bin2368@vip.163.com

Table S1. Oligonucleotides used in RT-qPCR

	Oligonucleotide 5′→3′ sequence	
	forward primers	reverse primers
16S rRNA	GGGTTTCGATACCCTTGG	GGGCTTTCAGAGGGATG
Gene 1	CAGACGAAGGTCGACAAG	GTGGAAGTGCATCTGGG
Gene 2	CTGAAGGCCGAAGAAGTATG	CTCCGGAATCCCGTACC
Gene 3	ATCCATCCGAAGGTTCAC	ACCGTATTGTACCAGATGC
Gene 4	AGAAATACGAACCTTATCTCACA	GTCGCCGTTCTTGATATTCAT
Gene 5	GTGAAGGAGGGCAAGTG	CGGTCATGAATTCGATGTC
